# Hepatitis B screening among Chinese Americans: a structural equation modeling analysis

**DOI:** 10.1186/s12879-015-0854-7

**Published:** 2015-03-08

**Authors:** Grace X Ma, Guo Yolanda Zhang, Shumenghui Zhai, Xiang Ma, Yin Tan, Steven E Shive, Min Qi Wang

**Affiliations:** Department of Public Health, Director of Center for Asian Health, College of Public Health, 1301 Cecil B Moore Ave. Ritter Annex, Rm 913, Philadelphia, PA 19122 USA; Center for Asian Health and College of Public Health, Temple University, Philadelphia, PA 19122 USA; Department of Health, Research Associate, Center for Asian Health, Temple University; and East Stroudsburg University, DeNike Hall, 200 Prospect St., East Stroudsburg University, East Stroudsburg, PA 18301-2999 USA; Department of Public and Community Health, University of Maryland, College Park, Maryland, USA

**Keywords:** HBV, Hepatitis B, HBV screening, Chinese, Health care access

## Abstract

**Background:**

Hepatitis B Virus (HBV) disproportionately affects new immigrants from endemic regions such as China. Untreated infections increase health risks for liver diseases including cancer. Yet most of those infected are unaware of their disease limiting prevention and early treatment options. The purpose of this community based study was to evaluate a heuristic model identifying factors contributing to Hepatitis B (HBV) screening among Chinese Americans.

**Methods:**

A cross-sectional design included a sample of 924 Chinese men and women 18 years of age and older of which 718 had complete data for final analysis. Confirmatory factor analysis verified conceptual indicators including access/satisfaction with health care and enabling, predisposing, cultural, and health belief factors. Structural equation modeling was used to identify direct and indirect predictors of Hepatitis B screening.

**Results:**

Bivariate analysis revealed that Chinese respondents who were never screened for HBV were significantly more likely to be below age 40 (69.8%), male (69.2%), had less than a high school education (76.4%), with less than 6 years living in the US (72.8%) and had no health insurance (79.2%). The final model identified enabling factors (having health insurance, a primary health care provider to go to when sick and more frequent visits to a doctor in the last year) as the strongest predictor of HBV screening (coefficient = 0.470, t = 7.618, p < .001). Predisposing factors (education variables) were also significantly related to HBV screening. Cultural factors and Satisfaction with Health care were associated with HBV screening only through their significant relationships with enabling factors.

**Conclusions:**

The tested theoretical model shows promise in predicting HBV testing among Chinese Americans. Increasing access to health care by expanding insurance options and improving culturally sensitivity in health systems are critical to reach new immigrants like Chinese for HBV screening. Yet such strategies are consistent with DHHS Action plan for the Prevention and Treatment of Viral Hepatitis. Implementing community-based strategies like partnering with relevant Community-Based Organizations are important for meeting HBV policy targets.

**Electronic supplementary material:**

The online version of this article (doi:10.1186/s12879-015-0854-7) contains supplementary material, which is available to authorized users.

## Background

An estimated 1.25 million people are chronically infected with hepatitis B virus (HBV) in the United States and 5000 die each year from HBV-related liver complications [1,2]. Chronic hepatitis infection causes 80% of all primary liver cancers with a low 5-year survival rate of only 10%. HBV remains the third most common cause of cancer death among Asians [2]. Approximately 10% of US Asians are infected with HBV, and the earlier in life an individual is infected, the higher the chances that he/she will become a chronic carrier [3,4]. HBV-related liver cancer rates among male Chinese Americans are 6 times higher than they are among Caucasians [5,6]. Asians in general and Chinese in particular are at greater absolute risk of HBV-related chronic diseases and death because the lifetime prevalence rate is higher among Asians already infected [1]. While HBV is spread through contact with blood, unprotected sex, shared needles, and from an infected mother to her newborn baby during delivery, Chinese are most likely to be infected as a newborn and may have HBV infection throughout their life. This early infection accounts for the large number of carriers in this and other Asian groups [2,3]. While tests can detect HBV and current medications extend life, Chinese Americans are generally unaware of the consequences of the disease [4]. Studies have shown that nearly half of this population (46%) have low knowledge of the relationship between HBV and liver cancer and have equally low rates of screening for HBV (35-48%) [7-9].

Educational, gender, English language proficiency, household income and knowledge about HBV are associated with HBV testing among Asians [10,11]. Other studies have shown that cultural factors affect HBV screening including respect for authority, elders and males in the decision-making process, ying/yang, chi and karma [12]. A strong belief in karma, for example, can lead Individuals to hide their HBV infection form family members [13]. Many Asians believe that talking about HBV infection may exacerbate the illness (e.g., bring bad luck to the infected person and others close to him or her) [14-17].

Decisions about HBV screening and vaccination in families are usually made by the father or eldest male offspring. While Asians are not likely to conceal their illness to a physician, a return to the same physician may be unlikely with patients opting instead, for a personal solution through ying/yang. Often Asian Americans use a combination of traditional medicine and Western medicine [18] but subgroup differences in the use of Western medicine still exist especially among recent immigrants [19,20].

**Figure 1:**
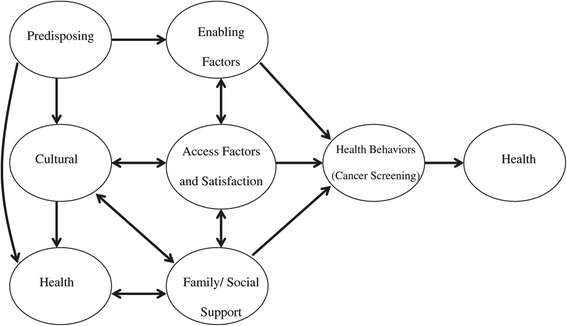
**Sociocultural Health Behavior Model.** Sociocultural Health Behavior Model, developed by the primary author, was used in this study to explore factors impacting hepatitis B screening and vaccination among the target population.

Figure 1 represents the Sociocultural Health Behavior Model (SCHBM) that identifies and describes relationships between, and interactions among the various factors that guide health behavior [21]. The SCHBM, a research-based model developed by the primary author, identifies six major factors that impact decision-making in health-seeking behaviors that lead to health care utilization. These include factors that affect health care utilization including: 1) predisposing (e.g., demographic and social characteristics), 2) cultural (e.g., health perceptions and beliefs, psychological status within the context of culture), 3) needs (e.g., urgency of care based on family health history, hierarchy of health care), 4) enabling (e.g., health coverage, communication, transportation), 5) environmental/health systems (e.g., barrier-free health care facilities, resource availability), and 6) family/social support (e.g., responsible and caring family and community). The SCHBM emphasizes the central and significant role that social-cultural factors have in influencing health behaviors.

The purpose of this community based study was to evaluate the SCHBM factors by developing a data based, heuristic model of concepts relevant to Hepatitis B (HBV) screening among Chinese Americans.

## Method

### Sample

This study, which focused on Chinese Americans, aged 18 and above and was part of a larger study which included four Asian American ethnic groups: Chinese, Korean, Vietnamese, and Cambodians. A regional Asian Community Cancer Coalition including the Center for Asian Health, Temple University identified 111 Asian community organizations in the greater Philadelphia (PA) area, New Jersey, and New York City. These organizations were located in geographic areas that maximized the coverage of Asian Americans across ethnic groups, ages, and socioeconomic status. Fifty-two organizations were randomly selected as clusters from the list and stratified based on the four ethnic and language groups. A proportional sampling procedure was adopted based on the size of each group with the exception of Cambodians which was oversampled because of its small size [22]. More than 2240 individuals were invited from the 52 sampled organizations to participate in the study, of which, 2098 were eligible and agreed to participate. Adults who met the critic of age 18+ and a 5th grade or higher level of reading proficiency in either English or their respective native language were eligible to participate in the study. Among the recruited eligible participants, 2011 (95.9%) completed the study. The final sample of 2011 consisted of 45.9% Chinese (n = 925); 19.1% Koreans (n = 384), 18.1% Vietnamese (n = 362), and 16.9% Cambodians (n = 340). Because list wise deletion of missing data eliminated many respondents subgroup analysis was not possible. Hence, Chinese respondents were the focus of the final analysis (analytic n = 718).

### Design and data collection

A cross-sectional research design was used in this study to provide point-in-time estimates of community attitudes and behaviors relevant to HBV screening with a representative sample [23,24]. Data collection was carried out between June 2005 and October 2006. Data collection and survey administration trainings were provided to all administrators and on-site bilingual interpreters. The research team, in collaboration with community leaders, recruited eligible Chinese participants through their respective community organizations. All participants read and signed informed consent forms to participate in the study. The survey was administered to participants in groups with available bilingual staff and trained volunteers to answer questions or translate as needed. Instructions for survey completion were read aloud both in English and Chinese prior to administration by research team members. Participants completed surveys by responding in English or Chinese. The questionnaire took about 25 minutes to complete. This described study (Protocol Number: 7118) was approved by Social and Behavioral Committee B, Institutional Review Board (IRB) of Temple University.

### Measurements

A multi-lingual questionnaire was developed in English, translated into Chinese and back-translated, and pilot-tested for reliability, validity, and cultural appropriateness [See Additional file 1].

### Hepatitis B screening

Respondents answered the question of whether or not they had been screened for the hepatitis B virus by answering no (coded 1) or yes (coded 2).

### Satisfaction with health care

Seven items assessed a respondent’s perceptions of their health care and doctors’ services. The response categories were on a 5-choice scale: “poor”, “fair”, “good”, “very good”, and “excellent”.

### Enabling factor

The enabling factor was measured using three questions: Do you currently have health insurance? Do you have a primary health care provider to go to when you are sick? How many times did you visit your current primary physician in the last 12 months? The response categories were a binary choice (no/yes) for the first two questions. The third question is an ordinal scale with four choices: “never”, “1-2 times”, “3-4 times”, and “5 or more times”.

### Predisposing factor

The predisposing factor measured the education level of the participants, including their highest grade of school completed and their years of education completed.

### Cultural factor

The cultural factor included participants’ English proficiency and their level of information seeking (Internet use). The response categories were “not at all” to “very well” for English speaking and a binary choice (no/yes) for Internet use.

### Cancer fear factor

The cancer fear factor reflects the fear of knowing a bad cancer test result and whether or not the participants felt embarrassment about cancer. The response categories were “no” or ”yes”.

### Statistical analysis

Chi-square and t-tests for differences in means were used to analyze demographic differences between those receiving HBV screening versus those who were not screened. Confirmatory Factor analysis with maximum likelihood estimation was used to verify variables associated with each concept in the SCHBM. Non-significant variables and variables with substantial missing data were eliminated with 89.1% of the sample retained for analysis. Structural Equation Modeling (SEM) was used for latent model analyses with Mplus software V7.1 [25]. Results are used to construct a path model using tetrachoric correlations that depicts relationships among exogenous variables (those variables with both emanating paths and receiving paths) and endogenous variables (those variables with mostly receiving paths) [26]. Model fit tests: Multiple fit was estimated with comparative fit indices (CFI), where the value of 0.90 or higher is considered acceptable; Tucker-Lewis Index (TLI), where the value of 0.90 or higher is considered acceptable; and the root mean square error of approximation (RMSEA), with the value below 0.08 indicating a good fit [27,28]. The total model R-Square indicates the percentage of explained variance in the dependent variable.

## Results

### Sample characteristics

Table 1 includes results for differences between those participants who received HBV screening (n = 272) and those who did not (n = 446 never screened). Younger respondents (18–39 years old) were more likely to be never-screened (69.78%) than those in the 40–64 (59.41%) and 65+ age groups (60.00%) (*χ*^2^ (1) = 6.10, p < .05). Among males, 69.20% reported never-screened compared with 57.24% of females (*χ*^2^ (1) = 10.48, p < .01). Of those with less than high school education, 76.44% reported never-screened compared to 58.06% with high school or higher education who reported never-screened (*χ*^2^ (1) = 18.78, p < .01). Of those without current health insurance, 79.17% reported never-screened compared with 53.62% of those with health insurance reporting never-screened (*χ*^2^ (1) =44.13, p < .01). Of those living in the US less than 6 years, only 20.9% were screened for HBV compared to around 40% of those living longer in the US (*χ*^2^ (1) = 11.43, p < .01). Marital status, employment status and annual household income were not significantly related to screening status (i.e. Table 1).

**Table 1:** **Demographics and liver cancer screening (percentages)**

**Variables**	**Patients (%)**		
	**Never-screened n = 446**	**Screened n = 272**	**p values**
Age Category	69.8	30.2	.048
18-39			
40-64	59.4	40.6	
65+	60.0	40.0	
Gender			.044
Male	69.2	30.8	
Female	57.2	42.8	
Current marital status			.287
Unmarried	65.7	34.3	
Married	61.2	38.8	
Highest degree			.000
<High school	76.4	23.6	
= > High school	58.1	41.9	
Employment status			.587
Employed	62.5	37.5	
Unemployed	60.4	39.6	
Annual Income			.543
<$10,000	64.3	35.7	
$10,000-$30,000	62.9	37.1	
>$30,000	58.7	41.3	
Current health insurance			0.000
No	79.2	20.8	
Yes	53.6	46.4	
Years Living in the US			0.000
Less than 6 years	72.8	20.9	
6-15 years	61.8	40.7	
16 or more years	56.3	43.7	

### Measurement model

The final, standardized factor loadings for the indicator variables are presented in Table 2. The overall scale Cronbach’s alpha was .85, a medium score. The t scores obtained for the coefficients were all significant except for one variable (Embarrassment/Shame). The magnitude of the factor loadings and their significance provided evidence to support the convergent validity of the indicators (i.e. Table 2).

**Table 2:** **Parameter estimates for the hypothesized measurement model**

**Construct & indictors**	**Standardized factor loading**
**Satisfaction with health care**	
Arrangements for making appointments for medical care	0.91
Length of time waiting to see doctor at the office	0.83
Length of time between making an appointment for care and visit	0.82
Overall, how would you rate care at your medical group?	0.79
Convenience of location of the doctor’s office	0.85
Access to medical care whenever needed	0.88
Quality of care from your physician	0.75
**Enabling factor**	
Currently have health insurance?	0.87
Have a primary health care provider to go to when you are sick?	0.98
Number of times visited current primary physician in the last 12 months	0.62
**Predisposing factor**	
What is the highest grade of school you completed?	0.53
How many years of education have you completed?	0.82
**Cultural factor**	
Do you often use the Internet for sources of information?	0.91
How well do you think you speak English?	0.93
Do you usually speak your native Asian language at home?	0.34
**Cancer fear factor**	
Fear of a getting a bad test result	0.91
Embarrassment/shame	0.65

### Structural model

**Figure 2:**
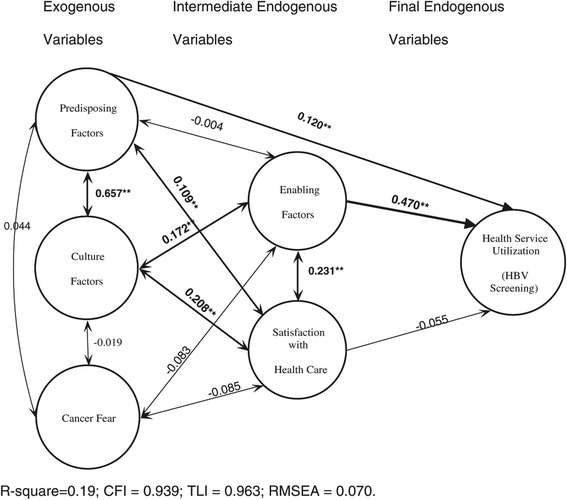
**Factors predicting HBV Screening with standardized (beta) SEM path coefficients (N = 718).** Final structural equation model using latent class analysis for the Sociocultural Health Behavior Model predicting hepatitis B screening and vaccination. Tetrachoric correlation and model goodness of fit tests were used to verify the direction and magnitude of associations between variable constructs and dependent variables.

The hypothesized, heuristic model with standardized maximum likelihood estimates for the parameters of the model are presented in Figure 2. The path coefficients indicate the direction and magnitude of the associations. The enabling factor (participants with health insurance, a primary health care provider, and frequent primary physician visits) showed a positive and significant relationship with HBV screening (coefficient = 0.470, t = 7.618, p < .001). The predisposing factor (education variables) also had a positive, significant relationship with HBV screening (coefficient = 0.120, t = 3.52, p < .001). Although the cultural factor was not directly related to screening, it was significantly related to the enabling and satisfaction with health care factors indicating that the influence of cultural factors was indirect via the enabling factor. The satisfaction with health care factor was not directly related to cancer screening. Overall, 19% of the hepatitis B liver cancer screening was explained by the model R^2^ and all indicators of goodness of fit were within acceptable ranges (i.e. Figure 2).

## Discussion

The purpose of this study was to validate a research-based Sociocultural Health Behavior Model (SCHBM) using Structural Equation Analysis (SEM) to illustrate the direction and magnitude of the SCHBM components in relation to HBV screening among Asian Americans. Among predisposing factors, age, gender, education, and health insurance status were significantly related to HBV screening. Younger persons, males, and those with less than a high school education were more likely to be “never screened” than if the persons who are older, female, and had more education. Although no previous studies explicitly looked at age, gender and education among US Asian Americans and Hepatitis B screening, reports of health fair events indicate older age (e.g. a mean age of 51) and females receiving HBV screening [29,30]. Reaching Asian American populations who are younger, of both genders and with lower education and/or literacy must be a priority for health agencies to improve prevention efforts and avoid HBV-related chronic disease and death [31].

The enabling factor was identified as the most important variable predicting HBV screening which primarily included measures of health insurance and access to a usual health care provider. Each indicator of enabling factors had virtually the same influence on HBV screening as variables were highly correlated to each other. Specifically only 20.8% of those without health insurance, 21.0% of those who had a physician to visit, and 17.6% of those who had not visited a doctor in the last year received HBV testing. The lack of health insurance leads to lower screening rates for a HBV and wide range of cancers [32]. Strategies identified in the US Initiative on Asian Americans and Pacific Islanders focuses on hepatitis and includes a national education campaign, working with community based organizations to develop outreach programs, disseminating successful interventions, training health care providers and improved data collection [1]. Missing in these strategies are mechanisms to improve access to health care including expanding health insurance options [33,34]. Despite recent health care reform, results from the National Health Interview Survey indicate that cancer screening rates will remain well below Healthy People 2020 targets, particularly for those with low income, no health insurance, and no usual source of care [35]. In order to stem the current HBV epidemic, health policy groups must consider new ways to increase health insurance for both HBV screening and for treatment.

Although the Satisfaction with Health Care factor was not directly related to HBV screening, it was significantly related to the Enabling factor and had an indirect coefficient of 0.109, almost twice as strong as the non-significant direct path (0.055). In past studies, health care satisfaction was highly related to health care utilization and, on average, Asians rated health care satisfaction lower than non-Hispanic whites [36-38]. Because these studies did not control for indirect effects such as having health insurance or a regular physician. It remains unknown whether or not the effects were moderated by enabling factors.

Cultural Factors did not have a direct effect on HBV screening but the combined coefficients for indirect influence on HBV Screening via the two endogenous variables, Enabling Factor and Satisfaction with Health Care, was moderate (0.103). Cancer Fear apparently had the least effect of any of the variables in the model. The newness of the SCHBM framework precludes direct comparison with other studies. However, past research supports the critical importance of barriers like language, literacy, and measures of acculturation like internet use [31]. These upstream factors are important barriers to health care utilization and vital components for public health approaches, especially when screening, vaccination and early treatment makes significant impacts on infectious diseases like HBV [1].

Predisposing Factor had both direct and indirect influences on HBV screening summing to a beta coefficient of 0.249, a larger effect than the direct effect (0.120). This suggests that there is a complex interplay between cultural factors, predisposing factors, satisfaction with Health Care and Enabling Factors as they explain health service utilization. From a model perspective, the strength of these combined relationships points to the importance of the SCHBM theoretical framework for understanding health behaviors embedded within cultural systems that are adapting to medical innovations such as new screenings and difficulties learning new systems that are common to recent immigrants. From an intervention perspective, these results suggest that effective interventions for Asian American populations must be multi-faceted and ecological. The comprehensive approach builds on Community Based Participatory Research principles [39]. Future research should further explore these concepts for other infectious diseases where screening and treatment are important for disease prevention, detection and management [1].

This is a community-based study aimed to understand the status of HBV screening and to identify barriers to screening in a community setting. As such, this study is limited insofar as the sample was recruited from participants connected to Community-Based Organizations and may not be representative of those who do are not so connected. In order to maximize participation, we did not request that participants allow us to validate their responses against medical records. Baseline studies of validated self-reports were not found for HBV screening. Available data for treatment adherence indicate that concordance between self-report and medical records could be as low as 70% and as high as 95% [40,41]. Missing data in this study also excluded approximately 12% of the sample, another limit. To date, no other studies were identified that tested theoretical models for understanding HBV screening in high risk populations. This study remains one of the first to test a theoretical model predicting HBV screening among Chinese Americans. In addition, the study findings provide a sound foundation for the development of culturally appropriate HBV screening interventions.

## Conclusions

The tested theoretical model shows promise in predicting HBV screening among Chinese Americans. Increasing access to health care by expanding insurance options and improving culturally sensitivity in health systems are critical to reach new immigrants like Chinese for HBV screening. Such strategies are consistent with DHHS Action plan for the Prevention and Treatment of Viral Hepatitis. Implementing community-based strategies like partnering with relevant Community-Based Organizations are important for meeting HBV policy targets.

## Additional file

Additional file 1:
**Hepatitis-B Client Survey, File: HBV_Client_Survey_ CAH.**

## Abbreviations

HBV: Hepatitis B virus
SCHBM: Sociocultural health behavior model
SEM: Structural equation modeling
CFI: Comparative fit indices
TLI: Tucker-Lewis index
RMSEA: Root mean square error of approximation

## References

[1] US HHS. HHS Plan for Asian American, Native Hawaiian and Pacific Islander (AANHPI) Health. Updated January 13, 2012. Accessed July 8, 2014. [http://minorityhealth.hhs.gov/templates/content.aspx?ID=8806&lvl=3&lvlid=573]

[2] Hepatitis B Foundation Statistics. Hepatitis B Foundation Website. Updated February 19, 2014. Accessed July 8, 2014. http://www.hepb.org/hepb/statistics.htm

[3] Hepatitis B Foundation Hepatitis B and primary liver cancer. Hepatitis B Foundation Website. Updated March 7, 2014. Accessed July 8, 2014. [http://www.hepb.org/professionals/hepb_and_liver_cancer.htm]

[4] Centers for Disease Control and Prevention Viral hepatitis surveillance – United States, 2010. Centers for Disease Control and Prevention Website. Updated May 24, 2013. Accessed July 8, 2014. [http://www.cdc.gov/hepatitis/statistics/2010surveillance/Commentary.htm]

[5] Nguyen MH, Keeffe EB (2003). Chronic hepatitis B and hepatitis C in Asian Americans. Rev Gastroenterol Disord.

[6] Miller BA, Kolonel LN, Bernstein L (1996). Racial/Ethnic patterns of cancer in the United States 1988–1992.

[7] Juon HS, Park BJ (2013). Effectiveness of a culturally integrated liver cancer education in improving HBV knowledge among Asian Americans. Prev Med.

[8] Taylor VM, Tu SP, Woodall E (2006). Hepatitis B knowledge and practices among Chinese immigrants to the United States. Asian Pac J Cancer Prev.

[9] Thompson MJ, Taylor VM, Jackson JC (2002). Hepatitis B knowledge and practices among Chinese American women in Seattle. Washington J Cancer Educ.

[10] Taylor VM, Choe JH, Yasui Y, Li L, Burke N, Jackson JC (2005). Hepatitis B awareness, testing, and knowledge among Vietnamese American men and women. J Community Health.

[11] Thompson MJ, Taylor VM, Yasui Y, Hislop TG, Jackson JC, Kuniyuki A (2003). Hepatitis B knowledge and practices among Chinese Canadian women in Vancouver, British Columbia. Can J Public Health.

[12] Hu KQ, Pan CQ, Goodwin D (2011). Barriers to screening for hepatitis B virus infection in Asian Americans. Dig Dis Sci.

[13] Ma GX, Lee S, Wang M, Tan Y, Gao W, Ma X (2011). Role of sociocultural factors in hepatitis B screening among Asian Americans. South Med J.

[14] Chen MS (1994). Keynote address of the seventh international conference on the health of Chinese in North America: health status of Chinese Americans: challenges and opportunities. Asian Am Pac Isl J Health.

[15] Dai YT, Dimond MF (1998). Filial piety. A cross-cultural comparison and its implications for the well-being of older parents. J Gerontol Nurs.

[16] Lassiter S (1995). Multicultural clients: a professional handbook for health care providers and social workers.

[17] McLaughlin LA, Braun KL (1998). Asian and Pacific islander cultural values: considerations for health care decision making. Health Soc Work.

[18] McCulloch M, Broffman M, Gao J, Colford JM (2002). Chinese herbal medicine and interferon in the treatment of chronic hepatitis B: a meta-analysis of randomized, controlled trials. Am J Public Health.

[19] Chan E, Tan M, Xin J, Sudarsanam S, Johnson DE (2010). Interactions between traditional Chinese medicines and Western. Curr Opin Drug Discov Devel.

[20] Ben-Arye E, Dipl PI, Baruch E, Dagash J (2014). Integrating family medicine and complementary medicine in cancer care: A cross-cultural perspective. Patient Educ Couns.

[21] Ma GX, Shive SE, Gao W, Tan Y, Wang MQ (2012). Prostate cancer screening among Chinese American men: a structural model. Am J Health Behav.

[22] Fowler FJ (2014). Survey research methods (Vol. 1).

[23] Babbie E (2012). The practice of social research.

[24] Israel BA, Eng E, Schults N, Parker EA (2013). Methods for community-based participatory research for health.

[25] Mplus [http://www.statmodel.com/] Accessed July 8, 2014.

[26] Loehlin JC (2004). Latent variable models: an introduction to factor, path, and structural equation analysis.

[27] Muthén B, Muthén LK (2000). Integrating person-centered and variable-centered analyses: growth mixture modeling with latent trajectory classes. Alcohol Clin Exp Res.

[28] Jöreskog KG (1993). Testing structural equation models. Sage focus editions.

[29] Nguyen K, Van Nguyen T, Shen D, Xia V, Tran D, Banh K (2014). Prevalence and presentation of hepatitis B and C virus (HBV and HCV) infection in Vietnamese Americans via serial community serologic testing. J Immigr Minor Health.

[30] Strong C, Hur K, Kim F, Pan J, Tran S, Juon HS (2015). Sociodemographic characteristics, knowledge and prevalence of viral hepatitis infection among Vietnamese Americans at community screenings. J Immigr Minor Health.

[31] Hwang JP, Roundtree AK, Suarez-Almazor ME (2012). Attitudes toward hepatitis B virus among Vietnamese, Chinese and Korean Americans in the Houston area. Texas J Community Health.

[32] Freund KM, Isabelle AP, Hanchate AD (2014). The impact of health insurance reform on insurance instability. J Health Care Poor Underserved.

[33] Green BB, Coronado GD, Devoe JE, Allison J (2014). Navigating the murky waters of colorectal cancer screening and health reform. Am J Public Health.

[34] Kapoor A, Battaglia TA, Isabelle AP (2014). The impact of insurance coverage during insurance reform on diagnostic resolution of cancer screening abnormalities. J Health Care Poor Underserved.

[35] Brown ML, Klabunde CN, Cronin KA, White MC, Richardson LC, McNeel TS: Challenges in meeting healthy people (2014). objectives for cancer-related preventive services, national health interview survey, 2008 and 2010. Prev Chronic Dis.

[36] Haviland MG, Morales LS, Reise SP, Hays RD (2003). Do health care ratings differ by race or ethnicity?. Jt Comm J Qual Saf.

[37] Lurie N, Zhan C, Sangl J, Bierman AS, Sekscenski ES (2003). Variation in racial and ethnic differences in consumer assessments of health care. Am J Manag Care.

[38] Murray-Garcia JL, Selby JV, Schmittdiel J, Grumbach K, Quesenberry CP (2000). Racial and ethnic differences in a patient survey: patients’ values, ratings, and reports regarding physician primary care performance in a large health maintenance organization. Med Care.

[39] Bell-Elkins J (2002). Assessing the CCPH principles of partnership in a community–campus partnership [Internet].

[40] Smith SR, Wahed AS, Kelley SS, Conjeevaram HS, Robuck PR, Fried MW (2007). Assessing the validity of self-reported medication adherence in hepatitis C treatment. Ann Pharmacother.

[41] Lawrence S, Morgan SG, Quan H (2012). Does Concordance between survey responses and administrative records differ by ethnicity for prescription medication?. J Popul Ther Clin Pharmacol.

