# Supplementary material for: Hepatitis B screening among Chinese Americans: a structural equation modeling analysis
Source: BMC Infect Dis. 2015 Mar 8;15:120. doi: 10.1186/s12879-015-0854-7 (PMC4357149; doi:10.1186/s12879-015-0854-7)
Supplement: Additional file 1: — Hepatitis-B Client Survey, File: HBV_Client_Survey_ CAH. [file 12879_2015_854_MOESM1_ESM.pdf]

Grace Ma, Ph.D., Principal Investigator,   Center for Asian Health, Temple University

Hepatitis-B Client Survey

1. Were you screened for Hepatitis B- Liver Cancer ?
_____ 1) I was never screened
_____ 2) Within last 12 months
_____ 3) Within 2.5 years
_____ 4) Over 5 years

2. What are the barriers that you have encountered when you taking screening for Hepatitis B? (**Check all barriers that apply**)

__________ 1) Lack of knowledge
__________ 2) I feel well, no health problems
__________ 3) Language problem
__________ 4) I do not have a regular doctor to see.
__________ 5) I have no time to do it
__________ 6) I did not know where to get services
__________ 7) No transportation to the facility
__________ 8) I have no insurance to cover the cost
__________ 9) My insurance does not cover the cost
__________ 10) Fear of a getting a bad test result
__________ 11) Embarrassment/Shame

3. Do you have a primary health care provider to go to when you are sick?
____ 1) No   _____ 2) Yes

4. Based on your experience with getting medical care, how would you rate the following?

	Poor	Fair	Good	Very Good	Excellent
1) Access to medical care whenever needed					
2) Arrangements for making appointments for medical care					
3) Length of time waiting to see doctor at the office					
4) Length of time you wait between making an appointment for care and the day of your visit					
5) Ability to contact a doctor after hours and on weekends					
6) Access to specialty care if you need it					
7) Access to medical care in an emergency					
8) Convenience of location of the doctor's office					
9) Hours when the doctor's office is open					
10) Availability of medical information or advice by phone					
11) Overall, how would you rate care at your medical group					
12) Quality of Care from your physician					

5. What year were you born? __________

6. What is your gender? _____1) Male ______2) Female

7. Were you born in the U.S? _____1) No ______2) Yes

8. How many years have you lived in the U.S.? ________ years

9. What is your current marital status?
____1) Married
____2) Never married
____3) Divorced/Separated
____4) Widower

10. How many years of education have you completed? _____ years.

What is the highest grade of school you completed? (**Check one below**)
____1) Non education or elementary school
____2) Below high school graduate
____3) High school
____4) University (or college or some college, or associate degree)
____5) Graduate and above

11. Which of the following describes you currently?
____1) Employed
____2) Unemployed
____3) Retired
____4) Homemaker
____5) Student

12. What is your annual household income?
____1) Less than $10,000
____2) $10,000-$20,000
____3) $20,000-$30,000
____4) $30,000-$40,000
____5) Above $40,000

13. Do you currently have health insurance? ____1) No ____ 2) Yes

14. Do you have a regular physician to visit? ____1) No ____2) Yes
(note, the article used "primary physician" instead of regular physician)

15. How well do you think you speak English?
____1) not at all ____2) not well ____3) well ____4) very well

16. Do you usually speak your native Asian language at home?
____1) No
____2) Yes (Circle one language: Chinese, Korean, Vietnamese, Cambodian, other_________)

17. Do you often use the Internet for sources of information? ______ 1) No   ______ 2) Yes
If your answer is Yes, which language do you primarily use:
____1) Chinese
____2) Vietnamese
____3) Korean
____4) Cambodian
____5) English
____6) Other (Please specify) _________

18. How many times have you visited your current primary physician in the last 12 months?
_____ 1) Never visited
_____ 2) 1-2 times
_____ 3) 3-4 times
_____ 4) 5 or more times
